# Dynamics of Volatile Compounds in Triticale Bread with Sourdough: From Flour to Bread †

**DOI:** 10.3390/foods9121837

**Published:** 2020-12-10

**Authors:** Ruta Galoburda, Evita Straumite, Martins Sabovics, Zanda Kruma

**Affiliations:** Faculty of Food Technology, Latvia University of Life Sciences and Technologies, Riga Street 22, LV-3001 Jelgava, Latvia; evita.straumite@llu.lv (E.S.); martins.sabovics@llu.lv (M.S.); zanda.kruma@llu.lv (Z.K.)

**Keywords:** volatile compounds, triticale, dough mixing and fermentation, bread crumb and crust

## Abstract

Triticale has been suggested for human consumption due to its valuable nutritional composition. The aim of this study was to evaluate volatile compound dynamics in the technological processes of triticale bread and triticale bread with sourdough prepared using *Lactobacillus sanfranciscensis* based cultures. Two types of sourdough ready-to-use sourdough and two-stage sourdough were used for bread making. Triticale bread without sourdough was used as a control. Volatile compounds from a headspace of flour blend, sourdough, as well as mixed dough, fermented dough, bread crumb and crust were extracted using solid-phase microextraction (SPME) in combination with gas chromatography/mass spectrometry. Alcohols, mainly 1-hexanol, were the main volatiles in the triticale flour blend, whereas in the headspace of sourdough samples ethyl-acetate, ethanol and acetic acid dominated. Two-stage sourdough after 30 min fermentation showed the highest sum of peak areas formed by 14 volatile compounds, resulting in substrates for further aroma development in bread. A total of 29 compounds were identified in the bread: in the crumb the dominant volatile compounds were alcohols, ketones, acids, but in the crust—alcohols, aldehydes, furans dominated. The use of two-stage sourdough provided a more diverse spectrum of volatile compounds. Such volatile compounds as ethanol, 3-methyl-1-butanol, 2-methyl-1-propanol, 2-hydroxy-2-butanone, 2-methylpropanoic acid, and acetic acid were identified in all the analysed samples in all stages of bread making.

## 1. Introduction

Production of triticale (x *Triticosecale* Wittmack) reached 12.8 million tonnes worldwide in 2018 and Europe is the major triticale producing region—89.7% of the global triticale production [1]. It has been mostly used as an animal feed. However, many studies have demonstrated its valuable nutritional composition [2,3] and health effects [4]. Triticale is characterised by a high amino acid–lysine content (0.31–0.71 g/100 g) and it allows the increase in fibre content in products because of its high content in the grains (11.7–13.6 g/100 g) [3,5]. Recent review suggests the potential of triticale as a complement of major cereals for various food applications [5,6]. Triticale may be used in the production of pastry, extruded products, pasta, and wheat-triticale bread. Although triticale is characterized by weak rheological properties, it is possible to produce good quality bread selecting acceptable varieties [7] and technologies [8,9]. Due to low gluten content and high alpha-amylase activity, weak baking properties hinder the use of triticale in bread production [3,10,11]. However, it is possible to make a product with the properties similar to those of wheat bread using flour blends, which incorporate other cereals such as triticale, oat, barley, sorghum by utilizing their starch gelatinization properties to form a wheat bread-like, aerated texture [12,13].

Sourdough technology may be used to improve bakery product quality [5], especially bread texture [14], nutritional and sensory attributes [15,16], and shelf life [17] and used as a natural antifungal agent in bread products [18]. It employs naturally occurring or selected lactic acid bacteria and yeasts. Sourdough fermentation requires a stable composition of starter cultures. Many researchers highlight the trend to use aroma-producing microorganisms for optimisation of the fermentation method, ensuring the improved quality of products, especially bread aroma [19,20]. Sourdough fermentation can decrease or increase the quantitative and qualitative amount of various compounds and affect the availability of nutrients [14,21]. The degradation of proteins is an important step in bread flavour development [22]. Proteolysis produces amino acids, which change the pH of dough and are precursors of aroma compounds [16]. The traditional sourdough preparation method requires daily refreshments to maintain activity of yeasts and lactic acid bacteria, which mainly is formed by heterofermentative *Lactobacilli* [23]. Recently, various semi-liquid preparations are developed by propagation. Another type of sourdough includes dried defined starter cultures.

Consumers pay special attention to the appearance, volume, colour, taste, and aroma of bread. Though, bread flavour is considered the most important attribute for consumer acceptability and product recognition [24]. Recently, it has been reported that different bread types can have up to 500 aroma compounds. Quantitatively the most important chemical classes forming bread flavour are the aldehydes, alcohols, ketones, esters, acids, pyrazines and pyrrolines, as well as other compounds such as hydrocarbons, furans, and lactones [25]. According to Pico et al. [26], the odour quality depends on many factors such as the recipe (flour and other ingredients) [3,27,28,29], the possible use of sourdough [30,31,32,33], enzymatic activity during dough mixing, the type of fermentation [30,34,35], the addition of enzymes [36,37] and improvers [17,38,39], and the reactions in the baking stage lipid oxidation and thermal reactions mainly through Maillard reactions and caramelisation [40,41]. During baking, due to varying temperatures in the inner and the outer part of the loaf, various volatile profiles are formed in the crumb and the crust [42]. The typical aroma of the bread crust is mainly produced in the baking process, while dough fermentation is the most important step in the development of crumb flavour.

Many studies have discussed the wheat and rye bread quality and composition of volatile compounds, but only little research was found on triticale bread [43]. Therefore, the aim of this study was to evaluate volatile compound dynamics in the technological processes of triticale bread and triticale bread with sourdough prepared using *Lactobacillus sanfranciscensis*-based cultures.

## 2. Materials and Methods

### 2.1. Flour and Other Ingredients

The triticale flour blend was made by mixing together flour of whole-grain triticale (60%), whole-grain rye (15%), whole-grain hull-less barley (15%), rice and maize (5% each). This composition was developed in earlier studies based on the dough rheological properties [44,45]. The triticale (variety “Inarta”), rye (variety “Kaupo”), as well as hull-less barley (variety “Irbe”) grain used in the study were bred in Latvia. The triticale, rye, and hull-less barley were ground in a laboratory mill Hawos (Hawos Kornmühlen GmbH, Hamburg, Germany). Rice and maize flour was purchased from Ustukiu Malunas, Ltd. (Pasvalys, Lithuania). The flour blend moisture content was 11.8%, protein content—8.75 g/100 g, total dietary fibre content—13.69 g/100 g, falling number—183 s. Baking properties of the flour blend: the water absorption—57.7%, the dough development time—4.74 min, the dough stability—7.10 min, the time to softening—12.25 min.

The following ingredients were included in the dough formulation: instant yeast (Sanf-instant, S.I.Lesaffre ‘Saninstant’, Maisons-Alfort, France), salt (Artimsol, Soledar, Donetsk region, Ukraina), and sugar (Dansukker, Nordic Sugar, Copenhagen, Denmark).

### 2.2. Preparation of Sourdough and Bread Making

Three types of bread were made for the study: triticale flour blend bread (FB-B) without sourdough; triticale flour blend bread with ready-to use sourdough *Sapore Fidelio* (SFS-B); and triticale flour blend bread with two-stage sourdough (TKS-B). Ready-to-use sourdough *Sapore Fidelio* (SFS) from Puratos (Groot-Bijgaarden, Belgium) was added 5% from flour amount. The second type of sourdough—two-stage sourdough (TKS) was made using flour blend and sourdough starter culture TK—Starter (Böcker, Minden, Germany), which contained rye milling products, water, glycerine, and sourdough culture (*Lactobacillus sanfranciscensis*). It was prepared according to the following procedure.

1. In the first stage of sourdough preparation, 10 g of starter culture was mixed with 100 g of flour blend, and 100 mL water (30 ± 1 °C). The dough after mixing was placed in a CL-53 thermostat (Memmert GmbH + Co. KG, Schwabach, Germany) for 24 ± 1 h at 28 ± 1 °C.
2. In the second stage, 100 g of flour blend and 100 mL water (30 ± 1 °C) was added to the sourdough made on the previous day. After thorough mixing it was placed in a thermostat for another 24 ± 1 h at 32 ± 1 °C. After the second stage, sourdough was ready for use in the bread preparation. The pH of ready TKS sourdough was 3.67.

The optimum technological parameters for triticale bread production were selected according to our previous studies [43]. Dough was mixed in a spiral type KM400 dough mixer (Kenwood Havant, Hampshire, UK) for 8 min (6 min—slow, 2 min—fast), dough temperature after mixing—25 °C. Samples were fermented and baked in the cast iron moulds. Dough was fermented for 10, 20 and 30 min at 35 °C at relative air humidity 80 ± 2% in a Sveba Dahlen proofer (Sveba Dahlen AB, Fristadt, Sweden). Samples after 30 min fermentation were baked in a Sveba Dahlen oven (Sveba Dahlen AB, Fristadt, Sweden) for 45 min at 200 ± 10 °C, steam was supplied for 3 s at the beginning of baking. Each bread type was made in two separate batches. The bread samples were analysed in triplicate after cooling to room temperature 22 ± 1 °C.

### 2.3. Determination of the Volatile Compounds

Volatile compounds from a headspace above the analysed samples of ingredients, dough, bread crumb and crust were extracted using solid phase microextraction (SPME). In the study the bipolar SMPE fibre with a Carboxen/Polymethylsiloxane (CAR/PDMS) coating of 85 µm thickness (Supelco Inc., Bellefonte, PA, USA) was used.

For the evaluation of volatiles present in flour blend, sourdough, and bread (crumb or crust), five grams of the respective sample were placed in a 20 mL vial. The extraction time was 65 min (incl., pre-incubation without fibre for 15 min) in a water bath at 40 °C.

Volatiles formed during mixing were adsorbed on a SPME fibre, which was inserted in a headspace of a dough mixer through a special hole (diameter 1 mm) as described by Sabovics et al. [46]. The volume of the mixer vessel was 4 litres. The sample size was based on 250 g of flour.

The detection of volatile compounds developed in the fermentation process was performed according to the method described by Sabovics et al. [47]. For detection of volatile compounds, a 250-mL glass container was equipped with the aluminium lid with a hole for the SPME fibre. The container was held in a Clifton Food Range water bath (Nickel-electro Ltd., Welton-S-Mare, Somerset, England) at water temperature 35 ± 1 °C for the fermentation process. The SPME extraction was done during whole fermentation process (10, 20, and 30 min).

Volatile compounds from fibre were thermally desorbed in the injector of a PerkinElmer 500 gas chromatograph - mass spectrometer (GC/MS) (PerkinElmer, Inc., Shelton, CT, USA) with a capillary column Elite-Wax ETR (60 m × 0.25 mm i.d.; DF 0.25 µm). For the GC/MS analysis the following parameters were set: the initial temperature 40 °C was held for 7 min, then it was increased from 40 °C to 160 °C at a rate of 6 °C/min, followed by the increase from 160 °C to 210 °C at a rate of 10 °C/min with the 15 min final holding time. Electron impact ionization mode was set at 70 eV, while the ion source and inlet line temperatures were both set to 250 °C. The carrier gas (helium) was supplied at a constant flow of 1 mL/min. Acquisition parameters were scanned at *m/z* 40–400. Compounds were identified by comparing their mass spectra with mass spectral library Nist98. Additionally, linear retention indexes were calculated based of the retention times of the alkanes (C8-C20) and were compared with data of the literature.

### 2.4. Analysis of Bread Physical-and-Chemical Parameters

The moisture content of the bread crumb was determined according to American Association of Cereal Chemists (AACC) method 44-15A. For pH measurement a Jenway pH-meter (Barloworld Scientific Ltd., Dunmow, Essex, UK) [43] was used. Water activity was measured according to standard ISO 21807:2004 by a LabSwift-aw device (Novasina AG, Lachen, Switzerland). Titratable acidity of the bread crumb was determined according to AACC method 02-31. Both bread crumb and crust colour were measured in the International Commission on Illumination (CIE) L*a*b* colour system by a Tristimulus Colorimeter (Accuracy Microsensors, Inc., Pitsford, NY, USA). Thehardness and stickiness of the bread crumb were evaluated using a TA.XT.plus texture analyser (Stable Micro Systems Ltd., Godalming, Surrey, UK) according to slightly modified AACC method 74-09.

### 2.5. Statistical Analysis

All acquired data are reported as means with standard deviation. One-way analysis of variance (ANOVA) was carried out to assess differences between means. Factors were estimated as significant if *p* ≤ 0.05. For determination of specific differences between samples, Tukey’s Post Hoc or a *t*-test were performed. The heat-map was obtained using free heat-mapping programme.

## 3. Results

### 3.1. Composition of Volatile Compounds in Raw Materials

**Figure 1:**
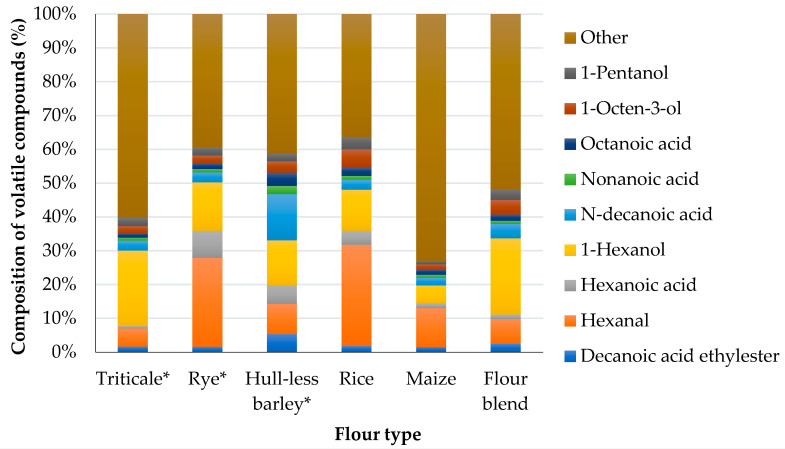
Composition of volatile compounds in flours and flour blend. * Whole-grain flour. (*n* = 3 for each type of flour).

In the analysed flours and flour blend, the variety of volatile compounds was detected (Figure 1). In whole-grain triticale flour 28 volatile compounds were identified, in whole-grain rye flour—24, in whole-grain hull-less barley flour—17, in rice flour—22, in maize flour—27. The quantitative composition of triticale flour blend was described by 31 volatile compounds. Among all identified the compounds the main chemical classes were: alcohols—13, carboxylic acids—6, terpenes—5, aldehydes and alkanes—4 compounds in each class, and esters—3 compounds.

In the rye and rice flour the highest peak areas were identified for hexanal, which is described as a fresh, green, grassy, leafy, fruity, sweaty scent [48]. Among all the studied flours, significantly smaller peak areas of hexanal were identified for triticale, hull-less barley, maize, and flour blend (Table S1, Supplementary Materials). The second most prolific compound was 1-hexanol, which adds a mild, sweet, alcohol, green grass, fruity sweet, woody and floral aroma [48], and comparing raw materials the highest proportion was detected in the whole-grain triticale and it resulted in its highest proportion in the flour blend.

The sourdough type showed differences in the spectrum of volatile compounds (Table 1). In TKS sourdough there were 11 volatile compounds identified, but in SFS sourdough—9 compounds. Five compounds were identified in both sourdough types—acetic acid, ethanol, ethyl acetate, 3-methyl-1-butanol, and isoamyl acetate.

In TKS the aromas were pineapple (ethyl acetate), alcohol (ethanol), sharp, acid-like (acetic acid), malt (3-methyl-1-butanol), sweet, fruit, pineapple, banana, malt, sharp and sour, green grass, greens (1-penten-3-ol), apple, anise (ethyl hexanoate), and honey (phenylethyl alcohol). In SFS the bouquet of aromas is composed of acid-like, sharp (acetic acid), sweet, pineapple (ethyl acetate), alcohol (ethanol), banana (isoamyl acetate), pear (butyl acetate), malt (3-methyl-1-butanol), and greens (hexanal).

### 3.2. Volatile Compounds in Mixed and Fermented Dough

During the bread production, in the mixing, fermenting, and baking processes changes occur of the physical-and-chemical parameters and various volatile compounds are formed in the bread crumb and crust [49]. Volatile compounds are produced through technological processes from flavour precursors present in cereal grains, such as amino acids, fatty acids and phenolic compounds [30]. Certain flavour–active volatile compounds, such as aldehydes, alcohols, and ketones are present in grain (Figure 1). According to the recently published studies, the use of sourdough provides better dough texture and higher loaf volume, as well enriching the bread aroma and taste [50,51]. Thus, the use of sourdough in the production of triticale bread may provide a specific taste and aroma of bread depending on sourdough type and production conditions.

#### 3.2.1. Volatile Compound Profile in Dough after Mixing

**Figure 2:**
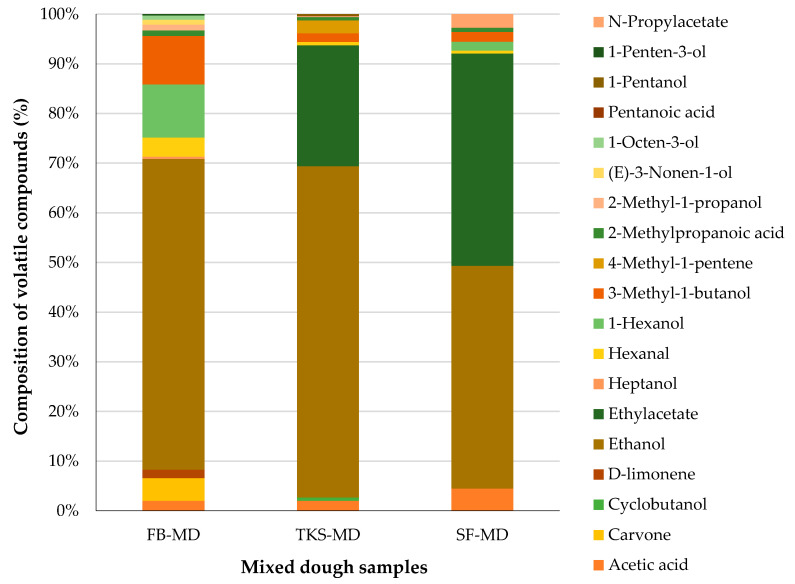
Percentage area of volatile compounds in the dough after 8 min mixing at dough temperature 25 ± 1 °C. (*n* = 3 for each type of dough). FB-MD: mixed flour blend dough, TKS-MD: mixed flour blend dough with two-stage sourdough, SFS-MD: mixed flour blend dough with ready-to-use sourdough.

In triticale flour blend dough and two types of dough with sourdough, 21 volatile compounds were identified after mixing (Figure 2). In triticale flour blend dough 13 were identified, in TKS-mixed dough (MD)—11, in SFS-MD—8 volatile compounds. The total peak area of volatiles detected in TKS and SFS dough was about 6.8-fold bigger compared to their area in dough made without sourdough.

Only four volatile compounds were common for all mixed dough samples, namely, ethanol, hexanal, 3-methyl-1-butanol, and acetic acid. Ethyl acetate, which added ethereal, fruity, sweet, and green aroma notes, was identified in both dough samples with sourdough as it was present in sourdough itself, but was not found in the flour blend. Cyclobutanol, 1-pentanol, 4-methyl-1-pentene, and pentanoic acid were detected only in TKS-MD. In turn, N-propylacetate was detected only in SFS-MD (Table S2, Supplementary Materials). Thus, it can be clearly seen that raw materials and their composition affect the development of volatile compounds in dough mixing. Additionally, Sabovics et al. [46] demonstrated that volatile compounds detected in mixed dough significantly differed at the same mixing times, changing dough temperatures (20, 25, 32 °C), except d-limonene. This can be related to the fact that the energy flow and the hydration are accompanied with the temperature increase. In bread making, the dough mixing contributes three main functions. First, homogenization blends the ingredients into a macroscopically homogenous mass. Second, air inclusion will form nuclei for gas bubbles that grow during dough fermentation. The third function of mixing in the dough structure formation is gluten development via mechanical energy input. During mixing substrates, which are useful for the generation of volatile compounds, are formed due to an increase in the enzymatic reactions, as well as non-enzymatic lipid oxidation which may increase as a result of incorporation of oxygen [49].

The main volatile compound in the mixed flour blend dough was ethanol, which gives dough a strong, alcohol, and medicinal aroma. In mixed flour blend dough without sourdough, 11% of all volatile compounds formed 1-hexanol, which can give dough ethereal, oily, alcohol, green, fruity sweet, woody, floral aroma notes, but in the TKS-MD and SFS-MD samples it was not detected.

#### 3.2.2. The Effect of Dough Fermentation on Volatile Compound Profile

**Figure 3:**
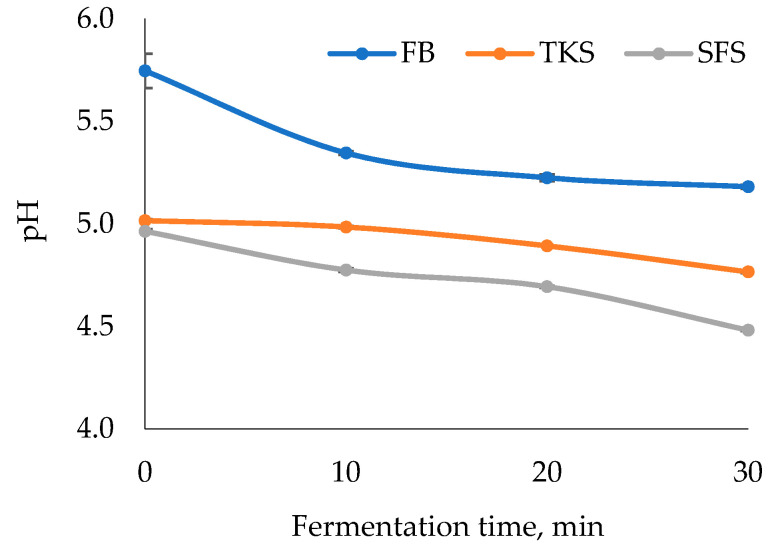
The dynamics of pH during triticale dough fermentation. Mean values with standard deviation bars (*n* = 6). FB: triticale flour-blend dough, TKS: two-stage sourdough, SFS: ready-to-use sourdough.

Volatile compounds were analysed after 10, 20 and 30 min of fermentation. The dynamics of pH in the fermentation process are presented in Figure 3.

**Figure 4:**
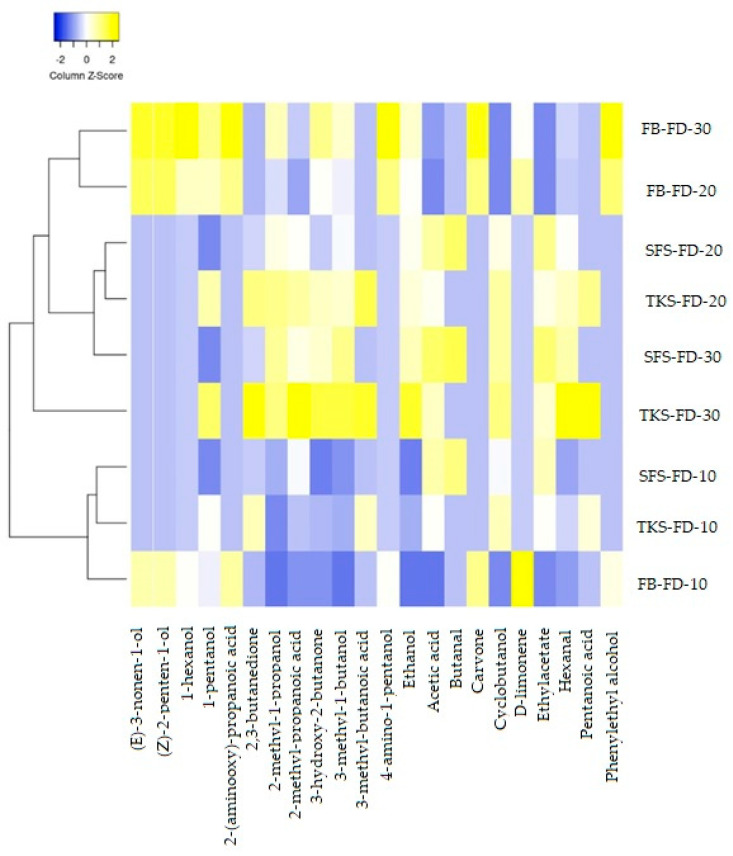
Heat-map of relative abundance and clustering results of the volatile compounds in dough depending on the use of sourdough and fermentation time. FB: triticale flour-blend dough, TKS: two-stage sourdough, SFS: ready-to-use sourdough, F10: fermentation time 10 min, F20: fermentation time 20 min, F30: fermentation time 30 min.

The results revealed significant difference in the volatile compound profile between the flour blend dough without sourdough and the triticale breads made with heterofermentative *Lactobacillus sanfranciscensis*-based sourdough. Totally, 22 volatile compounds were identified in the process of fermentation (Figure 4). Analysis of volatile compounds in fermented dough samples indicated that the peak areas of all volatile compounds increased with the fermentation time except d-limonene. The fermentation time and the sourdough used had a significant effect (*p* < 0.05) on the development of volatile compounds.

In all samples of the fermented dough, the highest peak areas were found for three alcohols (ethanol, 3-methyl-1-butanol, and 2-methyl-1-propanol) and one ketone–3-hydroxy-2-butanone. The highest areas of the above-mentioned alcohols and ketone were found in a dough with two-stage sourdough fermented for 20 (TKS-F20) and 30 (TKS-F30) minutes and with ready-to-use sourdough fermented for 30 min (SFS-F30). These volatile compounds are intensively formed as a result of alcoholic fermentation. They add the aroma notes of fruit, wine, whiskey, malt, burnt and green grass to the dough and fermented products. However, another two alcohols–1-hexanol and 4-amino-1-pentanol also showed high peak areas only in triticale dough (without sourdough) (FB-F20, FB-F30). Several alcohols were detected exceptionally in triticale dough (without sourdough), namely, alcohols ((Z)-2-penten-1-ol, (E)-3-nonen-1-ol, and phenylethyl alcohol), acids (2-methylpropanoic acid, 2-(aminooxy)-propanoic acid), and terpene (d-limonene). Their peak areas were less than 1 × 10^6^ and it was a very small amount compared to the other volatile compounds. As the time of fermentation increased, the peak area of carvone also increased but the peak area of d-limonene decreased, which could be explained by the fact that d-limonene can easily oxidize in moist air producing carveol, carvone, and limonene oxide [52]. Three volatile compounds (2,3-butanedione, 3-methyl-butanoic acid, and pentanoic acid) were detected only in dough made with two-stage sourdough. In turn, aldehyde butanal was specific only for dough made with ready-to-use sourdough.

Long-chain and complex alcohols can be produced in the metabolism of yeast, while alcohols can form aldehydes and ketones. Acetic acid is one of the main volatiles in sourdough and its formation continues during the yeast fermentation process and it adds acidic and vinegar aroma notes. The secondary metabolism of yeast can form 3-methyl-1-butanol, which gives a malty flavour to dough. According to Scieberle, [53] the amount and activity of yeast, dough fermentation time and temperature can affect the amount of flavour compounds formed in the dough.

All dough samples were grouped in four clusters according to the horizontal pattern of the heat-map based on the hierarchical clustering.

1. All three dough types after 10 min fermentation (FB-F10, TKS-F10, and SFS-F10). The cluster can be characterized by the smallest amount of volatile compounds.
2. Dough made with two-stage sourdough after 30 min fermentation (TKS-F30) was grouped separately from other samples. The highest sum of peak areas was detected in this sample. The volatile spectrum was formed by 14 volatile compounds, resulting in substrates for further aroma development in bread.
3. Dough made with ready-to-use sourdough after 20 and 30 min fermentation (SFS-F20, SFS-F30) and TKS-F20. The cluster with a medium sum of peak areas and small number of volatile compounds.
4. Triticale dough without sourdough after 20 and 30 min fermentation (FB-F20, FB-F30). The cluster with medium sum of peak areas and big number of volatile compounds, with a high intensity of (Z)-2-penten-1-ol, (E)-3-nonen-1-ol.

### 3.3. Triticale Bread Quality Parameters and Volatile Compounds in Its Crust and Crumb

#### 3.3.1. Physical-and-Chemical Characteristics of the Flour Blend Bread with Sourdough

The highest moisture content among the studied bread types was determined in the triticale bread with two-stage sourdough (Table 2). The bread pH was significantly decreased when sourdough fermentation was used in the dough development process.

The colour of crust and crumb of triticale bread with sourdough was light brown to brown. The sourdough used had no significant effect (*p* = 0.06) on the colour of triticale flour bread crust and crumb.

Two-stage sourdough had a significant effect (*p* = 0.02) on the hardness and stickiness of triticale flour blend bread crumb. The physical attributes of triticale bread with two-stage sourdough improved—the crumb of bread became softer and a little stickier, because the crumb had slightly higher moisture content compared to the bread without sourdough.

#### 3.3.2. Volatile Compounds in Crust and Crumb of Triticale Flour Blend Bread with Sourdough

In flour-blend bread without sourdough, in the crumb and crust 20 volatile compounds were detected (Table 3). The use of sourdoughs increased the variety of volatile compounds, resulting in 29 compounds detected in bread crumb and crust. Triticale bread (without sourdough) and bread with sourdough contained 19 common compounds, which probably are present due to the bread matrix. The sourdough used had a significant influence (*p* < 0.05) on the volatile compound qualitative and quantitative content in the crumb and crust of bread.

One compound (carvone) was specific to triticale bread (without sourdough), nine volatile compounds were specific to sourdough breads. Two compounds (pentanoic acid and one unidentified compound) were specific to triticale bread made with two-stage sourdough, which may result from the differences in the spectrum of the specific sourdough volatile compounds (Table 1). Butanoic acid peak area was much higher in the crust of bread made with ready-to-use sourdough compared to bread crust with two-stage sourdough. Peak areas of hexanal, 3-methyl-1-butanol, 2-methyl-1-propanol, 3-hydroxy-2-butanone, and 3-methyl-2-butanone were significantly higher in sourdough bread crumb compared to its crust. The majority of furans (2-furanmethanol, 2-methylfuran, and 5-methyl-2-furfural) were identified only in bread crust. A similar trend was observed for pyrrole.

When using SFS sourdough for triticale bread production, significantly more acetic acid, butanoic acid, and 3-methylbutanal were developed compared to other two bread types studied. The crust and crumb of triticale bread showed higher peak areas of volatile compounds when it was made with two-stage sourdough. In the crumb of bread the dominant volatile compounds were alcohols, ketones, acids but in the crust—aldehydes and furans.

In the sample SFS, ethanol formed 55.88% and 44.12% in the crumb and crust, respectively. The same distribution of other alcohols identified in the samples of bread crust and crumb was established. The use of two-stage sourdoughs in the current study allowed the obtaining more varied spectrum of volatile compounds, thus the bread had a more pronounced aroma.

### 3.4. The Dynamics of Volatile Compound Classes in the Technological Processes of Bread Production

**Figure 5:**
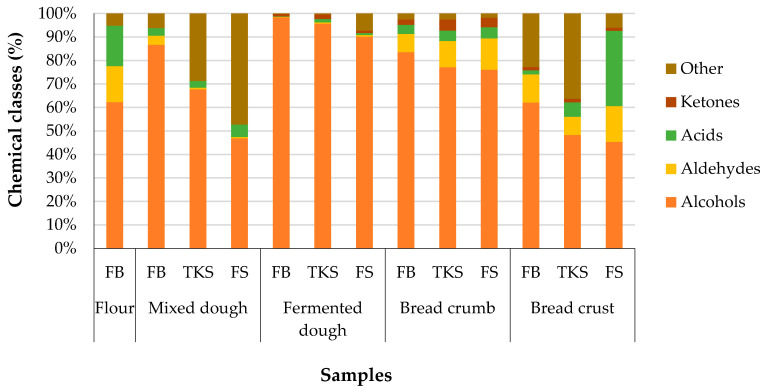
Dynamics of the volatile compound classes percentage in the bread production stages. FB: triticale flour blend; TKS: two-stage sourdough; SFS: ready to use sourdough.

Among the 32 identified volatile compounds only two (hexanal and 1-hexanol) were present at all stages of the triticale bread production. Alcohols, aldehydes and acids dominated in the spectrum of volatile compounds identified in flour blend (Figure 5). It also contained other compounds such as alkanes, terpenes and esters.

Analysis of the qualitative and quantitative contents of volatile compounds in the bread making process revealed that two compounds (1-octen-3-ol and heptanol) were identified exceptionally in the mixed dough. In dough with both ready-to-use (SFS) and two-stage sourdough (TKS) proportion of alcohols was significantly lower comparing to the FB sample, it is due to the main volatiles in sourdough—ethylactate (ester) and acetic acid. The spectrum of volatile compounds significantly differed (*p* < 0.05) in just mixed dough, fermented dough, and breadcrumb and crust providing specific aroma. According to Hansen and Schieberle [30], Pozo-Bayon et al. [56], Pico et al. [49] and others the aroma of bread is mainly formed during the fermenting and baking.

Alcohols are mainly formed during the fermentation process, what can be seen in triticale flour blend dough with the highest sum of alcohols relative areas. The highest proportion of alcohols was identified in the crust and crumb of triticale flour blend bread. The proportion of aldehydes in all analysed crust and crumb samples ranged from 7.5% to 15.2% and variation is significant but not very wide. Whereas the ketones proportion was significantly higher (*p* < 0.05) in bread crumb with TKS (4.7%) and SFS (4%) sourdough, in other bread samples it was lower than 2%. It is in agreement with other researchers who suggested that new volatile compounds are formed during baking. The formation of the volatiles in baking is based on the Maillard reaction—forming different alcohols, acids, aldehydes, furanes, ketones, pyrazines, and pyrroles [26,49]. The quantitative and qualitative profile of the mentioned compound classes significantly differed in bread crumb and crust. Various volatile compounds, such as aldehydes and ketones, are formed in the crust of bread in the range of 100 to 180 °C. During thermal processing, the number of alcohols decreased due to vaporization of moisture and alcohol. Alcohol 3-methyl-1-butanol, formed from amino acids due to the activity of yeast, plays a significant role in forming crumb and crust aroma.

The proportion of acids (2-methylpropanoic acid and acetic acid) during the process ranged from the mixed triticale dough to bread from 0.2% in fermented triticale flour blend dough up to 5.3% in mixed dough with SFS, excluding two extreme values—17% in FB flour and bread crust made with SFS, reaching 24.9%. SFS sourdough contained a significantly higher content of acetic acid compared to TKS sourdough.

## 4. Conclusions

The results revealed significant differences in the volatile compound profile between the flour blend dough without sourdough and the triticale breads made with heterofermentative *Lactobacillus sanfranciscensis*-based sourdough. The use of two-stage sourdough allowed the attainment of more diverse and wider spectrums of volatile compounds, and thus the bread had more pronounced aroma compared to triticale bread without sourdough. The use of ready-to-use sourdough resulted in triticale bread crust with a higher content of butanoic acid giving sharp, acetic aromas and 3-methylbutanal giving malty, roasty aromas. The volatile compound profile in the samples of triticale flour-blend dough and bread were different and in each stage of technological process, the newly formed volatile compounds were responsible for the aroma. Alcohols by far were the most important class of the volatile compounds during all bread technological processes. Although such volatile compounds as ethanol, 3-methyl-1-butanol, 2-methyl-1propanol, 2-hydroxy-2-butanone, 2-methylpropanoic acid, and acetic acid were identified in all analysed samples in all stages of bread making, giving the dough and bread fruity, malty, alcohol, buttery, creamy, and acid aromas.

## Figures and Tables

**Table 1:** The composition of sourdough volatile compounds relative peak areas (%).

Volatile Compounds	Sourdough Type	
	TKS	SFS
Acetic acid	23.36 ± 0.17 ^b^	48.15 ± 0.95 ^a^
Butyl acetate	n.d.	0.27 ± 0.03
Ethanol	32.16 ± 0.28	13.99 ± 0.61
Ethyl acetate	34.32 ± 0.42 ^a^	28.99 ± 0.61 ^b^
Ethyl hexonoate	0.14 ± 0.00	n.d.
Hexanal	n.d.	0.16 ± 0.00
Hexanoic acid	0.43 ± 0.03	n.d.
1-Hexanol	n.d.	4.91 ± 0.01
Isoamyl acetate	0.25 ± 0.01 ^b^	1.85 ± 0.04 ^a^
3-Methyl-1-butanol	1.60 ± 0.04 ^a^	1.25 ± 0.09 ^b^
4-Methyl-1-pentene	6.86 ± 0.05	n.d.
1-Pentanol	0.54 ± 0.01	n.d.
1-Penten-3-ol	0.11 ± 0.01	n.d.
Phenylethyl alcohol	0.23 ± 0.00	n.d.
N-Propylacetate	n.d.	0.43 ± 0.02
TOTAL	100.0	100.0

Mean values ± standard deviation (*n* = 3). TKS: two-stage sourdough; SFS: ready-to-use sourdough. n.d.—not detected. ^a,b^: Values with the different letters in the same row are significantly different, based on a *t*-test.

**Table 2:** Physical-and-chemical parameters of the triticale bread.

Parameters	Flour Blend Bread	Triticale Bread with Two-Stage Sourdough	Triticale Bread with *Sapore Fidelio* Sourdough
Moisture, %	43.74 ± 0.07 ^c^	45.13 ± 0.04 ^a^	44.17 ± 0.03 ^b^
Water activity	0.959 ± 0.001 ^a^	0.968 ± 0.001 ^a^	0.957 ± 0.001 ^a^
pH	5.196 ± 0.007 ^a^	4.642 ± 0.03 ^b^	4.377 ± 0.008 ^b^
Acidity, °	3.32 ± 0.04 ^c^	5.18 ±0.08 ^b^	6.02 ± 0.05 ^a^
Crumb colour			
L*	52.84 ± 0.57 ^a^	54.28 ± 0.53 ^a^	54.94 ± 0.65 ^a^
a*	0.61 ± 0.13 ^a^	0.59 ± 0.08 ^a^	0.53 ± 0.06 ^a^
b*	22.81 ± 0.55 ^a^	22.63 ± 0.68 ^a^	22.51 ± 0.77 ^a^
Crust colour			
L*	40.54 ± 0.70 ^a^	39.84 ± 0.77 ^a^	39.36 ± 0.69 ^a^
a*	4.41 ±0.13 ^a^	5.32 ± 0.11 ^a^	5.84 ± 0.47 ^a^
b*	22.72 ± 0.42 ^a^	20.34 ± 0.60 ^a^	19.97 ± 0.94 ^a^
Crumb hardness, N	14.53 ± 0.83 ^a^	12.05 ± 0.25 ^b^	14.01 ± 0.37 ^a^
Crumb stickiness, N	−0.56 ± 0.04 ^b^	−0.96 ± 0.04 ^a^	−0.60 ± 0.03 ^b^

Mean values ± standard deviation (*n* = 3). ^a–c^: different letters in a row indicate significant differences at *p* < 0.05 based on Tukey’s test.

**Table 3:** Volatile compound peak areas (×10^6^) in breadcrumb and crust, and their associated organoleptic characteristics.

Volatile Compounds	RT	LRI	Triticale Bread		TKS-B		SFS-B		Organoleptic Characteristic ^a,b,c,d,e,f^
			Crumb	Crust	Crumb	Crust	Crumb	Crust	
**Aldehydes**									
Heptanal	15.37	1204	13.40 ± 0.57	15.29 ± 0.40	15.14 ± 0.48	17.72 ± 0.08	13.58 ± 0.77	16.08 ± 0.57	Fresh, aldehydic, fatty, green, burgundy, ozone, grass
Hexanal	12.25	1103	17.26 ± 0.36	13.9 ± 0.17	34.98 ± 0.53	15.2 ± 0.38	25.6 ± 0.52	13.11 ± 0.10	Fresh, green, grass, fatty, aldehydic, leafy, fruity, flowery
3-Methylbutanal	7.01	926	5.7 ± 0.02	17.36 ± 0.11	13.8 ± 0.07	14.57 ± 0.62	29.07 ± 0.72	64.79 ± 0.90	Malty, roasty, cucumber-like
2-Methylpropanal	5.07	805	1.01 ± 0.01	6.25 ± 0.06	3.52 ± 0.24	11.23 ± 0.84	2.08 ± 0.10	8.64 ± 1.03	Wet cereal or straw
**Alcohols**									
Cyclobutanol	3.77	667	1.68 ± 0.05	3.54 ± 0.03	4.46 ± 0.07	5.91 ± 0.05	2.48 ± 0.05	4.01 ± 0.01	Roasted
Ethanol	7.29	942	214.97 ± 2.80	171.68 ± 2.37	286.71 ± 3.08	254.84 ± 0.09	259.16 ± 7.95	204.63 ± 5.98	Strong, alcohol, medicinal
1-Hexanol	19.99	1382	51.78 ± 054	17.76 ± 0.70	n.d.	n.d.	n.d.	11.79 ± 0.12	Ethereal, oil, alcohol, green, grass, fruity sweet, woody, floral
3-Methyl-1-butanol	16.22	1247	103.47 ± 0.75	43.72 ± 1.17	121.62 ± 2.83	58.58 ± 0.40	105.13 ± 2.80	49.38 ± 0.23	Oil, alcohol, fruity, banana, malty, almond, sweet, balsamic
2-Methyl-1-propanol (Isobutyl alcohol)	12.79	1209	12.4 ± 0.23	7.52 ± 0.11	12.65 ± 0.34	8.28 ± 0.27	11.76 ± 0.66	8.27 ± 0.20	Ethereal, winey, alcohol, malty
1-Pentanol	17.02	1306	n.d.	n.d.	14.83 ± 0.22	1.76 ± 0.12	2.07 ± 0.05	0.24 ± 0.01	Oil, sweet, balsamic, fruity
2-Phenylethyl alcohol	31.20	1738	13.03 ± 0.03	9.25 ± 0.40	28.56 ± 1.67	16.61 ± 0.72	14.86 ± 0.50	11.97 ± 0.98	Sweet, floral, fresh and bready with a honey nuance
**Ketones**									
3-Hydroxy-2-butanone (Acetoin)	18.71	1314	10.79 ± 0.08	6.36 ± 0.39	12.04 ± 1.01	7.01 ± 0.22	10.92 ± 096	7.01 ± 0.62	Sweet, butter, creamy, dairy product, caramel, fatty
3-Methyl-2-butanone	9.07	1001	n.d.	n.d.	16.46 ± 0.71	4.21 ± 0.22	10.74 ± 0.06	2.06 ± 0.04	Camphor
**Acids**									
Acetic acid	22.50	1454	7.7 ± 0.43	14.12 ± 1.04	7.13 ± 0.62	14.72 ± 0.504	9.41 ± 0.17	40.83 ± 1.67	Sharp, acrid, vinegar, sour
Butanoic acid (Butyric acid)	26.27	1573	n.d.	n.d.	0.76 ± 0.09	0.74 ± 0.01	1.31 ± 0.57	141.64 ± 3.06	Sharp, acetic, cheesy, butter, fruity, sweet, sour, sweat, Parmesan, rancid
Hexanoic acid (Caproic acid)	30.01	1698	3.1 ± 0.07	5.17 ± 0.07	7.93 ± 0.09	21.36 ± 0.13	5.07 ± 0.29	18.13 ± 1.09	Sour, fatty, sweat, cheesy, goat like
2-Methyl-propanoic acid (Isobutyric acid)	25.03	1532	3.06 ± 0.02	1.04 ± 0.08	2.13 ± 0.31	7.21 ± 0.18	2.97 ± 0.11	7.21 ± 0.05	Acidic, sour
3-Methyl-butanoic acid (Isovaleric acid)	27.07	1600	4.83 ± 0.16	n.d.	5.01 ± 0.08	1.13 ± 0.05	5.96 ± 0.09	7.22 ± 0.20	Parmesan cheese, sweaty, butter, fatty, sour, rancid
Pentanoic acid	29.29	1674	n.d.	n.d.	4.29 ± 0.35	1.6 ± 0.02	n.d.	n.d.	Stinky, putrid, acidic, sweat, rancid, acrid
**Heterocyclic Compounds**									
2-Furan methanol	27.05	1599	n.d.	71.4 ± 0.59	n.d.	141.43 ± 1.77	n.d.	1.3 ± 0.05	Alcohol, chemical, bread, coffee, musty, sweet, caramel, brunt, oil, earthy
Furfural (2-Furaldehyde)	23.01	1469	7.37 ± 0.09	13.32 ± 0.04	8.42 ± 1.99	46.73 ± 0.65	9.25 ± 0.02	6.57 ± 0.13	Sweet, woody, almond, bread, rancid, roasted, toasted
2-Methylfuran	17.87	1328	n.d.	n.d.	n.d.	33.13 ± 0.55	n.d.	22.77 ± 0.09	Ethereal, acetone, chocolate
5-Methyl-2-furfural	25.43	1546	n.d.	n.d.	n.d.	7.53 ± 0.18	n.d.	0.91 ± 0.10	Spicy, caramel, maple, almond
Maltol (3-Hydroxy-2-methyl-4-pyrone)	32.13	1768	4.56 ± 0.09	6.23 ± 0.13	8.84 ± 0.63	19.65 ± 1.75	5.3 ± 0.14	13.87 ± 1.37	Caramel, warmy-fruity
Pyrrole	28.15	1636	n.d.	n.d.	n.d.	10.96 ± 0.72	n.d.	12.09 ± 0.87	Nut
**Others**									
Carvone	28.62	1652	4.9 ± 0.05	6.13 ± 0.11	n.d.	n.d.	n.d.	n.d.	Mint, licorice
Tridecane	18.26	1338	n.d.	9.11 ± 0.09	n.d.	2.18 ± 0.04	n.d.	2.28 ± 0.24	Alkane
n.i.	23.94	1496	n.d.	n.d.	n.d.	12.74 ± 0.51	n.d.	6.51 ± 0.38	-
n.i.	35.16	1868	n.d.	n.d.	7.09 ± 0.13	20.46 ± 0.76	n.d.	n.d.	-

Mean values ± standard deviation (*n* = 3). n.d.: not detected, RT: retention time, min, LRI: calculated linear retention indexes based on the retention time of alkanes C8-C20, TKS-B: triticale bread with two-stage sourdough, SFS-B: triticale bread with ready-to-use sourdough, n.i.: not identified. ^a^—Petel et al. [48], ^b^—http://www.thegoodscentscompany.com, ^c^—www.flavornet.org, ^d^—Noir Qharul Izzreen et al. [54], ^e^—Parker [55], ^f^—Pico et al. [26].

## Supplementary Materials

The following are available online at https://www.mdpi.com/2304-8158/9/12/1837/s1, Table S1: Relative area of volatile compounds in flours and flour blend (%), Table S2: Relative area of volatile compounds in mixed dough.
