# Supplementary material for: Dynamics of Volatile Compounds in Triticale Bread with Sourdough: From Flour to Bread
Source: Foods. 2020 Dec 10;9(12):1837. doi: 10.3390/foods9121837 (PMC7763431; doi:10.3390/foods9121837)
Supplement: Supplementary file 1 [file foods-09-01837-s001.pdf]

Table S1. Relative area of volatile compounds in flours and flour blend (%)

Volatile compound	Triticale*	Rye*	Hull-less barley*	Rice	Maize	Flour blend
Decanoic acid ethylester	1.65 ± 0.07 c	1.59 ± 0.07 c	5.34 ± 0.04 a	1.84 ± 0.06 c	1.56 ± 0.06 c	2.48 ± 0.18 b
Hexanal	5.43 ± 0.04 d	26.29 ± 0.72 a	8.97 ± 0.30 c	29.95 ± 0.63 a	11.50 ± 0.32 b	7.35 ± 0.22 c
Hexanoic acid	0.67 ± 0.02 e	8.04 ± 0.27 a	5.60 ± 0.30 b	4.11 ± 0.32 c	1.42 ± 0.03 d	1.29 ± 0.06 d
1-Hexanol	22.34 ± 0.76 a	14.34 ± 0.32 b	13.21 ± 0.50 bc	12.17 ± 0.37 c	5.27 ± 0.29 d	22.57 ± 0.37 a
N-decanoic acid	2.96 ± 0.16 c	2.97 ± 0.20 c	13.64 ± 0.89 a	2.98 ± 0.17 c	2.25 ± 0.03 d	4.27 ± 0.11 b
Nonanoic acid	0.84 ± 0.02 c	0.98 ± 0.02 bc	2.42 ± 0.08 a	1.12 ± 0.05 b	0.93 ± 0.02 bc	0.87 ± 0.03 c
Octanoic acid	1.27 ± 0.03 d	1.57 ± 0.08 c	3.95 ± 0.34 a	2.41 ± 0.05 b	1.50 ± 0.04 c	1.83 ± 0.12 c
1-Octen-3-ol	2.13 ± 0.26 de	2.42 ± 0.22 d	3.39 ± 0.04 c	5.48 ± 0.20 a	1.61 ± 0.02 e	4.36 ± 0.10 b
1-Pentanol	2.44 ± 0.31 b	2.40 ± 0.13 b	2.23 ± 0.03 b	3.64 ± 0.07 a	1.03 ± 0.05 c	3.00 ± 0.16 a
Other	60.27 ± 1.67 b	39.39 ± 1.82 d	41.25 ± 2.52 d	36.30 ± 1.93 e	72.93 ± 0.86 a	51.97 ± 1.35 c

* whole grain flour. All data are means ± standard deviation (n = 3). a, b, c … : values with the different lowercase letters in the same row are significantly different, based on Tukey’s test.

Table S2. Relative area of volatile compounds in mixed dough

Volatile compounds	FB-MD	TKS-MD	SF-MD
Acetic acid	2.02 ± 0.14 b	2.03 ± 0.03 b	4.47 ± 0.15 a
Carvone	4.57 ± 0.21	n.d.	n.d.
Cyclobutanol	n.d.	0.68 ± 0.04	n.d.
D-limonene	1.70 ± 0.08	n.d.	n.d.
Ethanol	62.62 ± 0.48 a	66.63 ± 3.24 a	44.88 ± 0.10 b
Ethylacetate	n.d.	24.36 ± 1.50 b	42.73 ± 0.09 a
Heptanol	0.44 ± 0.10	n.d.	n.d.
Hexanal	3.85 ± 0.01 a	0.64 ± 0.02 b	0.59 ± 0.01 b
1-Hexanol	10.70 ± 0.15	n.d.	1.79 ± 0.03
3-Methyl-1-butanol	9.78 ± 0.16 a	1.74 ± 0.03 c	1.95 ± 0.01 b
4-Methyl-1-pentene	n.d.	2.62 ± 0.12	n.d.
2-Methylpropanoic acid	1.11 ± 0.05	0.65 ± 0.1 b	0.89 ± 0.08 a
2-Methyl-1-propanol	1.17 ± 0.25 a	0.20 ± 0.07 b	n.d.
(E)-3-Nonen-1-ol	0.98 ± 0.06	n.d.	n.d.
1-Octen-3-ol	0.85 ± 0.14	n.d.	n.d.
Pentanoic acid	n.d.	0.17 ± 0.01	n.d.
1-Pentanol	n.d.	0.28 ± 0.01	n.d.
1-Penten-3-ol	0.26 ± 0.05	n.d.	n.d.
N-Propylacetate	n.d.	n.d.	2.70 ± 0.12

All data are means ± standard deviation (n = 3). FB-MD: mixed triticale flour blend bread; TKS-MD: mixed flour blend dough with two-stage sourdough; SF-MD: mixed flour blend with ready-to-use sourdough. n.d.: not detected. a, b, c: values with the different letters in the same row are significantly different, based on Tukey's or t-test.
